# Quality Change in Camellia Oil during Intermittent Frying

**DOI:** 10.3390/foods11244047

**Published:** 2022-12-14

**Authors:** Xiaofang Liu, Shuo Wang, Yong Yu, Xu Zhang, Jieyu Chen, Han Zhang

**Affiliations:** 1 School of Tourism and Cuisine, Yangzhou University, Yangzhou 225127, China; 2 School of Food Science and Engineering, Yangzhou University, Yangzhou 225127, China; 3 Faculty of Bioresource Sciences, Akita Prefectural University, Akita 010-0195, Japan

**Keywords:** camellia oil, frying, unsaturated fatty acid, tocopherol, quality, volatile compound, principal component analysis

## Abstract

Camellia oil with a high oleic acid content is widely used for frying. To comprehensively describe the quality change in camellia oil during frying, the changes in composition, deterioration indicators, and volatile profiles were investigated. The results showed that tocopherols mainly degraded in the early stage of frying, followed by unsaturated fatty acids (UFA). This caused the carbonyl value and total polar compounds level to significantly increase. Moreover, frying promoted the accumulation of volatile compounds in terms of type and abundance, especially aldehydes, which are related to the degradation of UFA. Principal component analysis showed that the frying of camellia oil was divided into three stages. First, the camellia oil with a heating time of 2.5–7.5 h showed excellent quality, where tocopherol played a major role in preventing the loss of UFA and was in the degradation acceleration stage. Subsequently, as tocopherol entered the degradation deceleration stage, the quality of camellia oil heated for 10.0–15.0 h presented a transition from good to deteriorated. Finally, tocopherol entered the degradation stagnation stage, and the quality of camellia oil heated for 17.5–25.0 h gradually deteriorated, accompanied by a high level of volatile compounds and deterioration indicators. Overall, this work comprehensively determined the deterioration of camellia oil during intermittent frying and offered valuable insights for its quality evaluation.

## 1. Introduction

Camellia oil is an edible vegetable oil extracted from mature camellia seeds (*Camellia oleifera* Abel). It abounds in Asia, especially in southern China [1]. Camellia oil is honored as “oriental olive oil” [2], because it has similar fatty acid composition and some trace functional substances (i.e., squalene, phytosterols, polyphenols, and tocopherols) to olive oil [3]. These beneficial compounds in camellia oil have been proven to have the effects of lowering blood pressure and cholesterol, protecting the liver and cardiovascular system, and anti-oxidation [4,5,6]. As a result, the use of camellia oil in the diet, especially in fried foods, is believed to be beneficial to health care, making it increasingly popular all over the world.

With the widespread consumption of camellia oil, its quality has been widely emphasized. The existing research focuses on the following aspects, including adulteration detection and quality changes in the process of refining, storage, and use [7,8,9]. Generally, camellia oil with high oleic acid was conducive to high oxidation stability and was suitable for being frying oil. However, the diversity and complexity of physicochemical changes during frying would significantly affect the quality of camellia oil. To date, research on the quality changes in camellia oil during frying has received scant attention [10,11].

Traditionally, the quality of frying oil was evaluated through deterioration indicators [12,13,14]. An excellent chemical indicator for measuring the degradation of frying oil is the carbonyl compounds value (CV), which is the total amount of carbonyl compounds in the secondary oxidation products formed throughout the process of oil degradation [15]. China and Japan have stipulated a CV limit of 50 μmol/g for frying oil [16]. Furthermore, the frying process also results in the production of alcohol, acid, ester, and other polar compounds by oxidation, hydrolysis, isomerization, and polymerization. The quantity of these substances is not only related to food materials, frying conditions, and environmental factors [17], but also to the deterioration of the oil. These substances are known as total polar compounds (TPC), which is a representative indicator to describe the deterioration degree of oil. It has been widely used as the limit indicator of oil by various countries, among which Japan has the lowest TPC limit at 24% [16]. Recently, the relationships between the generation of volatile compounds and the deterioration of frying oil were largely investigated [18,19]. It was found that volatile compounds are not only primarily responsible for the overall flavor of edible oil [20], but also an important quality indicator for edible oil. Consequently, volatile compounds are also considered to be necessary for the evaluation of camellia oil quality. To specify the quality changes in camellia oil during frying and make up for the deficiencies in this field, the combination of compositional characteristics, deterioration indicators, and volatile compounds was believed to highlight the comprehensive understanding of what internal factors drive the deterioration of camellia oil and how these drivers change over time.

The present work describes the changes in fatty acid composition, tocopherol content, CV, TPC, and volatile compounds of camellia oil over time during frying. Subsequently, principal component analysis (PCA) was performed to comprehensively investigate the quality changes of camellia oil during frying from different aspects, including basic composition, deterioration indicators, and flavor profiles. Although this study is unable to encompass the quality changes of various types of camellia oil during frying, it still can provide valuable insights into the quality changes in camellia oil during frying.

## 2. Materials and Methods

### 2.1. Chemicals and Samples

Standard reagents, namely, fatty acid methyl standards, Vitamin E reference standards (d-α-, β-, γ-, and δ-tocopherol), 2,2,5,7,8-pentamethyl-6-hydroxychroman, and (*E*)-2-decenal, were purchased from FUJIFILM Wako Pure Chemical Corporation (Osaka, Japan). A custom alkane blend standard of C6–C16 was obtained from Restek Corporation (Bellefonte, PA, USA). Hydrochloric acid was purchased from Kanto Chemical Company Limited (Tokyo, Japan). Hexane, methanol, acetic acid, isopropanol, butylated hydroxytoluene, potassium hydroxide, 2,4-dinitrophenylhydrazine (2,4-DNPH), and *n*-butanol were purchased from FUJIFILM Wako Pure Chemical Corporation. Among them, hexane and isopropanol were chromatography grade and all other chemicals were analytical grade.

The camellia oil and frozen par-fried French fries were purchased in the local market (Akita, Japan). The frying process involved frying French fries at 180 °C in a restaurant-style stainless steel electric fryer (TF-20A, Taiji & Company Limited, Kanagawa, Japan) using three liters of oil. The temperature of the frying oil was kept constant at 180 °C during frying, and 75 g of French fries were fried for 3 min at 27 min intervals. This operation was repeated for 5 h per day for 5 consecutive days, with 150 mL of frying oil taken every 2.5 h, without adding additional oil to the fryer. Oil samples were stored at −18 °C before analysis.

### 2.2. Determination of Fatty Acid Composition

A capillary gas chromatograph (GC-2010, Shimadzu Company, Kyoto, Japan) was used for the qualitative and quantitative determination of fatty acids in oil samples and reported as relative area percentage. First, the oil samples (0.1 g) used in the analysis were methyl esterified by adding methanolic potassium hydroxide solution (0.1 mL, 2 M) and vortexing in hexane (10 mL). Gas chromatography with an HP-88 capillary column (100 m length, 0.25 mm internal diameter, 0.2 μm film thickness) and a flame ionization detector was used to identify samples. The column temperature was set to rise from 120 °C to 170 °C at a rate of 10 °C/min, then to 250 °C at a rate of 4 °C/min, where it remained for 5 min. The detector temperature was adjusted to 260 °C. Using an AOC-20i auto-injector with a split ratio of 1:30, a sample (1 mL) was injected. The peaks of fatty acid methyl esters were determined by comparing the retention times of the samples with those of the standards [21].

### 2.3. Determination of Tocopherol Content

Tocopherols were determined following our previous study [22]. The oil sample was dissolved into 10 mL of hexane and passed through a 0.20-μm membrane filter after the addition of the internal standard 2,2,5,7,8-pentamethyl-6-hydroxychroman. High-performance liquid chromatography (Shimadzu, Kyoto, Japan) equipped with a normal-phase Shodex silica 5SIL 4D analytical column (150 mm × 4.6 mm i.d., Showa Denko K.K., Tokyo, Japan) was used for analysis. The column oven was set at 40 °C. A fluorescence detector was used for detection with an excitation wavelength of 298 nm and an emission wavelength of 325 nm. The mobile phase consisted of hexane, isopropanol, and acetic acid (1000:6:5, V:V:V) and contained 5 mg/mL of butylated hydroxytoluene at a flow rate of 0.8 mL/min. The calibration curves of α-, β-, γ-, and δ-tocopherol were created separately to quantify the content of each tocopherol.

### 2.4. Determination of Carbonyl Compounds Value

The CV of the oil samples was determined by adopting the Japan Oil Chemists’ Society Official Method Tentative 13-2013 [23]. Briefly, the oil sample was weighed and dissolved in *n*-butanol (10 mL) in a volumetric flask. The 2,4-DNPH (50 mg) was dissolved in 100 mL of *n*-butanol that also contained 3.5 mL of concentrated hydrochloric acid to create a 2,4-DNPH solution. A potassium hydroxide solution was prepared by dissolving potassium hydroxide (8 g) in 100 mL *n*-butanol. (*E*)-2-Decanal standard solutions were prepared by dissolving in *n*-butanol and diluting to concentrations of 100, 200, and 400 μmol/L. The analytes (1 mL) were mixed with 1 mL of 2,4-DNPH solution and placed in a 40 °C water bath for 20 min, then cooled to room temperature with tap water. Then, 8 mL of potassium hydroxide solution was added, mixed well, and centrifuged at 3000 rpm for 5 min. The absorbance of the samples was measured using a spectrophotometer (Benchmark Plus Microplate Reader, Bio-Rad, Tokyo, Japan) at 420 nm and quantified with the standard curve.

### 2.5. Determination of Total Polar Compounds

According to the manufacturer’s instructions, the TPC level of hot oil was rapidly determined using a food oil monitor (FOM 320, Ebro Electronics, Ingolstadt, Germany). The monitoring sensor was first immersed in hot oil with the optimum operating temperature between 150 °C and 180 °C. The oil was gently stirred for 20 s to ensure an even temperature distribution. The sensor was left in the hot oil until the TPC value and temperature on the monitor screen stabilized. This determination was repeated at least three times to ensure that the temperature was stable within ± 2 °C. The most frequent stable value of TPC was recorded [24].

### 2.6. Analysis of Volatile Compounds

The analysis of volatile compounds was performed on a headspace-gas chromatography/mass spectrometry system (QP2020, Shimadzu, Kyoto, Japan). The oil sample of about 1.000 g was introduced in a 20 mL vial, sealed with a magnetic cap. The vial was equilibrated for 30 min at 80 °C. After equilibrium, 1 μL of the sample was injected at a split ratio of 1:10. Helium was used as carrier gas at a flow rate of 11 mL/min. Chromatographic separation was achieved on an SH-Rxi-5Sil MS capillary column (30 m × 0.25 mm i.d. × 0.25 μm film thickness, Shimadzu). The oven temperature program began at 40 °C, held for 5 min, raised at 4 °C/min to 250 °C, and kept for 3 min. The ion source and interface temperatures were set at 200 and 230 °C, respectively. To determine the retention times and characteristic mass fragments, the mass spectra of the samples were recorded in full scan mode, ranging from 35 to 350 *m/z*.

The qualitative analysis of volatile compounds was carried out by matching the mass spectrum with the standard spectra provided by the National Institute of Standards and Technology (NIST17) database (Agilent Technologies Inc., Gaithersburg, MD, USA). The qualitative results were verified by matching the Kováts retention indices (RI) of volatile compounds with the SH-Rxi-5Sil MS capillary column or DB-5 column reported in the literature. The RI was calculated by using C6–C16 *n*-alkanes with the Van den Dool equation as follows [25]:

(1) $$\mathrm{RI}=100\times \left(n+\frac{{\mathrm{RT}}_{i}-{\mathrm{RT}}_{n}}{{\mathrm{RT}}_{n+1}-{\mathrm{RT}}_{n}}\right)$$

where RT*_i_* is the retention time of a certain unknown volatile compound to be measured, RT*_n_* < RT*_i_* < RT*_n+_*_1_, and the subscripts of *n* and *n* + 1 are the carbon-atom numbers of *n*-alkanes before and after the appearance of the certain unknown volatile compound in GC/MS.

A semi-quantitative analysis was adopted for the quantification of the identified volatile compounds, that is, the peak area of the sample with standard quality is used for quantification, which is obtained by dividing the measured peak area by the weight of the sample.

### 2.7. Statistical Analysis

All the analysis was performed in triplicate and the results obtained were expressed as mean ± standard deviation. Analysis of variance (ANOVA) was used for performing data analysis. The variance among the samples at a significance level of *p* < 0.05 was determined by applying Duncan’s multiple range test. All statistical analyses (ANOVA, regression, correlation, and PCA) were performed using SPSS software version 26.0 (SPSS Inc., Chicago, IL, USA).

## 3. Results and Discussion

### 3.1. Changes in Fatty Acid Composition during Frying

The fatty acid composition of camellia oil was analyzed and shown in Table 1. Five predominant fatty acids were detected in camellia oil, namely palmitic acid, stearic acid, oleic acid, linoleic acid, and linolenic acid. The total unsaturated fatty acids (TUFA) proportion was as high as 91.45%, with monounsaturated oleic acid accounting for the largest contribution (78.42%), and polyunsaturated fatty acids (PUFA) accounting for only 13.04%. Similar results for camellia oils were reported by Zhu et al. [26] and Wang et al. [27].

The dynamic change in the fatty acid composition of camellia oil during frying was also shown in Table 1. The degradation of unsaturated fatty acids increased due to the instability of double bonds as frying proceeded, resulting in a decrease in the proportion of unsaturated fatty acids and an increase in the proportion of saturated fatty acids. The change in fatty acids showed a linear relationship with time, and the fitting results are shown in Table 2. The slope represents the change rate of fatty acids with time, where a large value of slope represents a rapid change. A positive value indicates the proportion of fatty acid increases during frying, while a negative value indicates consumption during frying. It can be seen that the change rate of palmitic acid was the fastest, and its increase rate was 1.5 times that of stearic acid. This was not only related to the decrease in the proportion of unsaturated fatty acids, but also attributed to the interaction between camellia oil and palmitic acid of par-fried French fries, because most French fries in the industry use palm oil for pre-frying [28]. For unsaturated fatty acids, the decrease rate of linolenic acid was faster than that of linoleic acid, and the two were much faster than oleic acid, depending on the number of their double bonds.

It also can be found from Table 1 that the change in fatty acid profile was sharper in the later frying stage (15.0–25.0 h) compared with that in the early frying stage. Considering that antioxidants are also important internal factors affecting the oxidation rate of oil, relevant research was carried out to explore the impact of antioxidants on the difference in the loss rate of unsaturated fatty acids in different frying stages, and then reveal the impact of antioxidants on the quality of the oil.

### 3.2. Changes in Tocopherol Content during Frying

As the representative of the most common antioxidant in oil, tocopherols are important nutrients and play key roles in the quality maintenance of vegetable oils [29]. They can play an antioxidant role through various biochemical and biophysical mechanisms, including scavenging reactive oxygen and free radicals and acting as an effective chain terminator in lipid oxidation reactions [30]. The content of α-, β-, γ-, and δ-tocopherol was measured in the present study and shown in Table 3. It was found that the tocopherol composition of camellia oil was mainly α-tocopherol (8.19 mg/100 g), followed by γ-tocopherol (4.39 mg/100 g), and the content of β- (0.06 mg/100 g) and δ-tocopherol (0.13 mg/100 g) was much lower than that of the former two tocopherols. These tocopherols may be conducive to maintaining the oxidative stability of camellia oil.

The change in tocopherol content with the frying process was determined. The results showed that the content of the four tocopherols gradually decreased with time. The decreasing rate of tocopherols was different in the order of α > γ > δ, and β-tocopherol is not discussed further in the present work because of its low content and low antioxidant activity [31]. Among tocopherols, Yanishlieva et al. [32] reported that α-tocopherol was more active through a kinetic model, indicating that the hydrogen radical of α-tocopherol reacted more quickly with the fatty acid chain. According to the present results, we speculate that α-tocopherol in camellia oil has the highest antioxidant activity during frying, which has an important contribution to quality and safety.

In addition, it was also found that the loss rate of tocopherols gradually increased with time, and the loss rate in the early frying stage (0.0–12.5 h) was 93.23%, much faster than that in the later stage. Meanwhile, it was noted that the faster loss of tocopherols resulting in the slower loss of unsaturated fatty acids; while tocopherols had no statistically significant change in the later stage of frying, entering a degradation stagnation stage, and then the unsaturated fatty acids were rapidly lost. This could be attributed to the antioxidant effects of tocopherols, which can slow down the oxidation of unsaturated fatty acids at the early frying stage and maintain oil quality. Conversely, the content of tocopherols greatly decreased with continuous consumption, and the competition for the oxidation of unsaturated fatty acids was strengthened, leading to the acceleration of the deterioration of camellia oil in the later stage. Consequently, the important roles played by tocopherols in maintaining the quality and safety of camellia oil were demonstrated.

### 3.3. Evolution of Carbonyl Value and Total Polar Compounds Level during Frying

**Figure 1:**
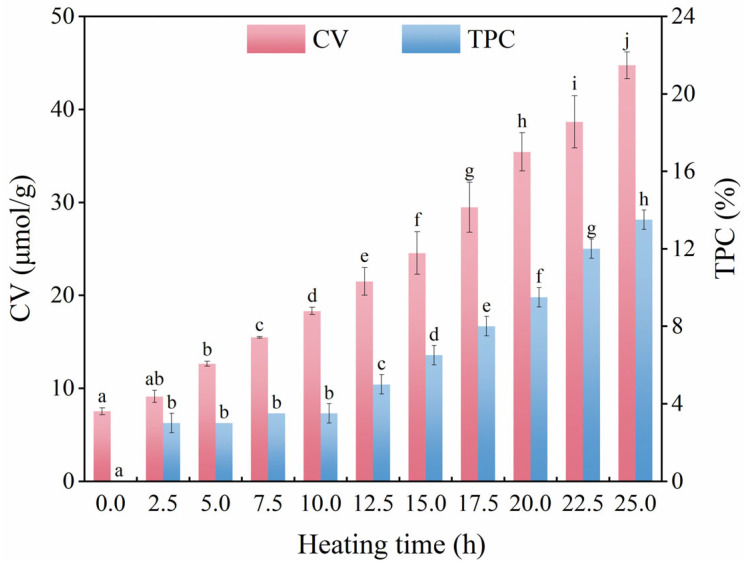
Change in carbonyl value (CV) and total polar compounds (TPC) level during frying. Different superscript letters in the same color column indicate significant differences (*p* < 0.05).

Before frying, the initial CV of camellia oil was 7.52 μmol/g. The variation in CV with time during the frying process is shown in Figure 1. As shown, the CV of camellia oil gradually increased throughout the frying process. Moreover, linearity between the CV and heating time was determined with a high determination coefficient (R^2^ = 0.966):

CV = 1.33*t* + 7.52 (2)

where *t* (h) is the heating time.

It was observed that after 25 h of frying and heating, CV reached a maximum value of 44.72 μmol/g, and the quality of camellia oil was still within the national safe use limit (50 μmol/g). Taking CV as the deterioration indicator, the frying life of camellia oil obtained by extrapolation was 31.94 h. However, it should be noted that in the actual frying process, a larger frying container will lead to an increase in the contact surface between oil and air, and further, the oxidation degree of frying oil is sharper than in the present experiment. For safety, the actual frying life of camellia oil in the frying process should be less than 31.94 h, and an available limit for frying life needs more detailed information.

The TPC level of camellia oil before frying was below the detection limit, and the changes in the TPC level of camellia oil during frying are shown in Figure 1. As shown in Figure 1, it was found that there was a slight increase in TPC within 10.0 h, and a steady increase after 10.0 h until the end. At the end of frying, TPC reached a maximum level of 13.5%, which was still within the safe use limit. Overall, TPC showed an upward trend with time, and the linear fitting equation with a determination coefficient of 0.941 was as follows:

TPC = 0.49*t* (3)

In addition, compared with TPC, the change in CV was considered to be more sensitive and feasible due to the more obvious dynamic trend in the frying process. By far, when camellia oil is used for intermittent frying under the same frying conditions, the CV was selected as the deterioration indicator and the frying life of camellia oil was suggested to be controlled within 31.94 h.

### 3.4. Changes in Volatile Compounds Profiles

The volatile compounds in camellia oil during frying were identified to reveal the difference in flavor and, in turn, differences in quality. There were ten volatile compounds identified in the camellia oil before frying, including two alcohols, four aldehydes, two esters, and two alkanes (Table 4). The profile changed dramatically when the oil was exposed to frying, and the type of volatile compounds identified increased to 32 after frying, including four new alcohols, 14 new aldehydes, and four new other volatile compounds, indicating that heat treatment would significantly promote the formation of volatile compounds, especially for aldehydes.

The volatile compounds in oils were divided into three chemical categories according to properties and quantity, namely alcohols, aldehydes, and other compounds. Alcohols usually come from the degradation of fatty acid hydroperoxides [33]. Alcohols contribute to green, fruit, and acid aroma notes in the sensory evaluation of camellia oil [34]. Among these alcohols, the abundance of ethanol was the highest before frying. Similarly, Multari et al. [35] also detected 73.1% of ethanol in oat oil dominated by oleic acid, which may be related to the autoxidation of oil. The abundance of ethanol decreased sharply once fried and then increased gradually with the extension of heating time. The abundance of 2-methylpropan-1-ol was significantly increased and was the highest after frying. Pent-1-en-3-ol, pentan-1-ol, heptan-1-ol, and octan-1-ol were absent in the oil before frying, but were detected after frying. Their abundance increased with heating time. Molina-Garcia et al. [36] also found that these alcohols were not detected before frying but detected after frying in extra-virgin olive, peanut, and canola oils with low PUFA. As a result, these alcohols are believed to be generated by the thermal oxidation of unsaturated fatty acids, which requires a certain amount of energy.

Aldehydes are either direct products of unsaturated fatty acid oxidation or reaction products of oxidation intermediates [37]. In the present study, aldehydes were the most-detected volatile compounds. The saturated aldehydes, butanal, 2-methylbutanal, pentanal, and hexanal, were naturally present in the oil before frying. The massive enrichment of these volatile compounds during frying may be related to their lower formation enthalpies [38]. It was found that the abundance of these compounds fluctuated with time (Table 4). This dynamic variation could be attributed to their boiling points being lower than the frying temperature, resulting in the dynamic competition between generation and volatilization. Among them, pentanal and hexanal were the most abundant aldehydes, mainly due to their diverse formation pathways. Pentanal can be formed by linoleic acid and (2*E*,4*E*)-deca-2,4-dienal, while hexanal can be formed from the degradation of linoleic acid, oleic acid, and other unsaturated aldehydes, including (2*E*,4*E*)-deca-2,4-dienal [37,39]. Overall, these saturated aldehydes (butanal, pentanal, hexanal, heptanal, octanal, and nonanal) are related to the aromas of fat, green, and citrus that contribute to the flavor profile of camellia oil [40].

As for unsaturated aldehydes, they were all absent in camellia oil before frying, but detected after frying, indicating that unsaturated aldehydes were produced during frying. The present study identified a total of 11 unsaturated aldehydes, which were the most diverse of all volatile compounds. Among these unsaturated aldehydes, (*E*)-but-2-enal, (*E*)-pent-2-enal, (*E*)-hex-2-enal, (*E*)-hept-2-enal, (*E*)-oct-2-enal, (*E*)-non-2-enal, (*E*)-dec-2-enal, and (*E*)-undec-2-enal were assigned to alkenals, while (2*E*,4*E*)-hepta-2,4-dienal, (2*E*,4*E*)-deca-2,4-dienal, and (2*E*,4*E*)-undeca-2,4-dienal were assigned to alkadienals. It was found that the alkenals increased gradually with the decrease in unsaturated fatty acids during frying, indicating that the degradation of unsaturated fatty acids contributed to the generation of alkenals. It has also been reported that (*E*)-but-2-enal, (*E*)-pent-2-enal, and (*E*)-hex-2-enal are oxidation products of linolenic acid and its oxidation intermediates; (*E*)-hex-2-enal, (*E*)-oct-2-enal, and (*E*)-non-2-enal are oxidation products of linoleic acid; while (*E*)-dec-2-enal and (*E*)-undec-2-enal are derived from the oxidation of oleic acid [37]. The present results are in good accordance with the previous report. Furthermore, the abundance of the alkenals consisting of four to seven carbon atoms was larger than that of other alkenals. This could be attributed to the differences in their generation pathways: the short-chain alkenals with four to seven carbon atoms are not only directly produced by unsaturated fatty acids, but also formed by the cleavage of long-chain alkenes or alkadienals during frying. In the case of alkadienals, (2*E*,4*E*)-hepta-2,4-dienal had slight but statistically significant fluctuations during frying. (2*E*,4*E*)-Deca-2,4-dienal was the peroxidation product from linoleic acid and showed the lowest abundance among alkadienals in the present study. This may be caused by the further utilization of the generated (2*E*,4*E*)-deca-2,4-dienal in the formation of pentanal and hexanal. It has a characteristic deep-fried aroma note and thus is an important aroma compound for oil and fried food [41]. In addition, the toxicity of aldehydes also has been extensively studied. It is believed that the toxicity of unsaturated aldehydes is more harmful than saturated aldehydes, of which alkadienals are the most toxic [42]. The toxicity difference of different aldehydes is mainly related to their hydrophobicity and electrophilicity [43]. It has been confirmed that (2*E*,4*E*)-hepta-2,4-dienal and (2*E*,4*E*)-deca-2,4-dienal have cytotoxicity and the latter also has genotoxicity [36,44]. Although the abundance of alkadienals detected in this study was less, considering safety, future research should try to avoid the formation of these unsaturated aldehydes during frying to prevent their migration from camellia oil to French fries or being ingested by humans. The safety and limited value of aldehydes are still a direction worthy of research in the future.

Besides aldehydes and alcohols, other volatile compounds including esters, acids, alkanes, furans, and pyrazines were also investigated. Esters are mainly formed by the esterification of alcohols with small-molecule free fatty acids. Two esters were identified in camellia oil: methyl acetate and ethyl acetate, which were detected before frying and irregularly fluctuated during frying. They are naturally present in camellia seeds and usually provide a sweet and fruity aroma [45]. The occurrence of three alkanes (i.e., hexane, heptane, and butylcyclopentane) in frying oils results from free radical reactions of unsaturated and saturated fatty acyl chains [46]. Among them, hexane increased significantly during frying, whereas heptane and butylcyclopentane showed a fluctuating trend. Moreover, it was observed that acetic acid, 2-pentylfuran, and 2-ethyl-6-methylpyrazine, which were absent in camellia oil before frying, showed a significant increase during frying. This indicated that these three compounds were positively generated during the thermal oxidation of camellia oil, and similar results were obtained by He et al. [2]. In detail, the generation of 2-pentylfuran is related to the shearing effect and intramolecular cyclization of linoleic acid. 2-Pentylfuran is considered to have an off-flavor in camellia oil [47]. 2-Ethyl-6-methylpyrazine is a heterocyclic compound that is produced by the Maillard reaction of L-aspartic acid and L-ascorbic acid in French fries during frying [48], with a roasted and nutty flavor [49].

**Figure 2:**
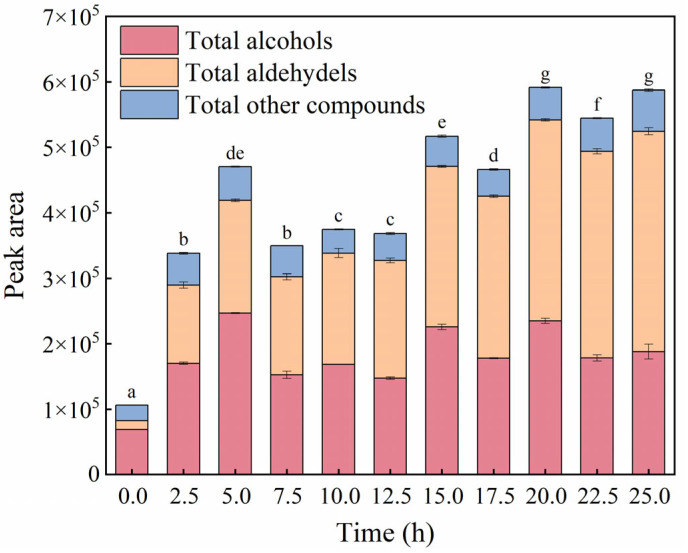
Change in the total peak area of aldehyde, alcohol, and other compounds identified in oil samples with different heating times. Different superscript letters indicate significant differences in the total peak area of all volatile compounds (*p* < 0.05).

The general varied trend of volatile compounds in three chemical categories with heating time is presented in Figure 2. Unheated camellia oil presented a volatile profile characteristic of a high abundance of alcohols, indicating that the formation of alcohols may be related to the automatic oxidation of camellia oil. After frying, the thermal degradation of unsaturated fatty acids leads to the formation of alcohols and aldehydes, especially the production of 14 new aldehydes, which markedly increases the abundance of aldehydes. The abundance of alcohols reached a maximum value at a heating time of 5.0 h and then fluctuated. This was because the smoke point of camellia oil gradually decreased with frying [50], which made the volatile compounds more likely to volatilize with the oil fume while continuously generating. In contrast, the decomposition of unsaturated fatty acids was more inclined to the accumulation of aldehydes, and the accumulation rate was greater than its volatilization rate. As a result, the abundance of aldehydes increased with time during frying and gradually exceeded that of alcohols after 15.0 h of heating. Consequently, alcohols were mainly responsible for the volatile composition of frying oils during the initial stage of frying (<15.0 h), after which aldehyde gradually dominated. The other volatile compounds fluctuated and slightly increased throughout the frying process with a significant difference.

In addition, the abundance of aldehydes was positively correlated with CV, with a correlation coefficient of 0.94 (*p* < 0.01). This indicated that the increase in the CV of camellia oil during frying was mainly contributed by aldehydes. The total abundance of all volatile compounds had a significant positive correlation with TPC, with a correlation coefficient of 0.85 (*p* < 0.01). Overall, the abundance and type of volatile compounds increased significantly during frying, indicating that frying had a significant impact on the generation of volatile compounds in camellia oil. The initial fresh fragrance of camellia oil eventually evolved into off-flavors, such as fat, grass, and rancidity, with the quality of the oil gradually deteriorating.

### 3.5. Principal Component Analysis of Quality Change in Oil

**Figure 3:**
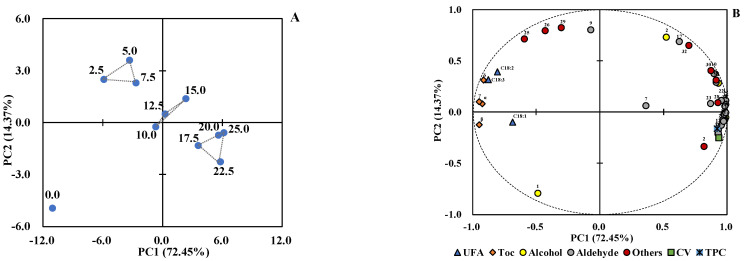
Principal component analysis (PCA) of unsaturated fatty acid (UFA), tocopherol (Toc), volatile compounds, carbonyl value (CV), and total polar compounds (TPC) level in the oil samples with different heating times. (**A**) Score plot; (**B**) Loading plot. The numbering of compounds in (**B**) is the same as the numbering of compounds in Table 4. C18:1, C18:2, and C18:3 represent oleic acid, linoleic acid, and linolenic acid, respectively.

PCA was performed to highlight the differences in oils and determine which factors contribute greatly to the quality change in oil during frying. The unsaturated fatty acids, tocopherol, CV, TPC, and volatile compounds were collected as a data matrix. The PCA results showed that the cumulative variance contribution rate of the first two principal components (PC1 and PC2) was 86.82%, where PC1 accounted for 72.45% and PC2 accounted for 14.37%. It is generally believed that the principal components whose cumulative variance contribution rate is greater than 85% can sufficiently explain the variation [51]. Therefore, PC1 and PC2 were selected to explain the regularity and difference between oils with different heating times (Figure 3).

The PCA score plot (Figure 3A) clearly showed that the quality of oils before frying and after frying were well distinguished in a relatively independent space. From the negative direction to the positive direction of PC1, the quality of oils gradually deteriorated. In addition, oils with different heating times were well clustered and separated: the oils with a heating time of 2.5–7.5 h were gathered into group 1 (G1), which was distributed in the second quadrant; the oils with a heating time of 10.0–15.0 h were gathered into group 2 (G2) and located almost at the origin of coordinates; the oils with a heating time of 17.5–25.0 h clustered into group 3 (G3) and were located in the fourth quadrant. The separation indicated that there were significant differences in the quality of oils with different heating times. In addition, referring to the results obtained by Xu et al. [41], the volatile characteristics of camellia oil can be divided into three distinct stages during frying: break-in, optimum, and degrading. The obtained PCA results, which clearly distinguish unheated camellia oil and frying camellia oils in G1, G2, and G3, are believed to show a similar effect on describing different quality characteristics for the frying process of camellia oil.

According to the loadings plot (Figure 3B), these variables not only contribute to PCA differentiation but also play a decisive role in the quality change of camellia oil during frying. It can be seen that unsaturated fatty acids and tocopherols had negative loadings on PC1, whereas CV, TPC, and most volatile compounds possessed positive loadings on PC1, indicating their different roles in the frying process of camellia oil. Specifically, G1 had a negative score on PC1 and a positive score on PC2, indicating the corresponding loadings (unsaturated fatty acids, tocopherols, 2-methylpropan-1-ol, 2-methylbutanal, acetic acid methyl ester, ethyl acetate, and heptane) made a great contribution to determining the quality of camellia oils with a heating time of 2.5–7.5 h. The quality of camellia oil in this stage was excellent, and its most important characteristics were the high content of unsaturated fatty acids and tocopherols. This stage was the main stage for tocopherol to play an antioxidant role, thus representing a degradation acceleration stage of tocopherol with a loss rate of 55.19%. The distribution of G2 showed that camellia oils with a heating time of 10.0–15.0 h achieved a state of equilibrium with all factors. G2 was the transition stage between G1 and G3, representing a quality change in camellia oil from good to deteriorated. Combined with Table 3, this stage was the degradation deceleration stage of tocopherol. The distribution of G3 suggested that the quality of oils in G3 could be characterized by five alcohols (ethanol, pent-1-en-3-ol, pentan-1-ol, heptan-1-ol, and octan-1-ol), fifteen aldehydes ((*E*)-but-2-enal, pentanal, (*E*)-pent-2-enal, hexanal, (*E*)-hex-2-enal, heptanal, (*E*)-hept-2-enal, octanal, (*E*)-oct-2-enal, nonanal, (*E*)-non-2-enal, (*E*)-dec-2-enal, (2*E*,4*E*)-deca-2,4-dienal, (*E*)-undec-2-enal, and (2*E*,4*E*)-undeca-2,4-dienal), and four other compounds (acetic acid, hexane, butylcyclopentane, and 2-ethyl-6-methylpyrazine), as well as CV and TPC. It also implied that the accumulation of these volatile compounds was beneficial to the high level of CV and TPC, as well as related to the quality deterioration in camellia oil during frying, which was the reason why G3 was defined as “degrading”. At this stage, tocopherol was almost consumed, entering the degradation stagnation stage. This was consistent with the above results that oils with a heating time of 17.5–25.0 h had a high abundance of volatile compounds, CV, and TPC. In addition, the distribution of camellia oil before frying (0.0 h) was far away from other frying oils (Figure 3A) because it was positively correlated with tocopherols and unsaturated fatty acids and negatively correlated with volatile compounds, CV, and TPC (Figure 3B), which can be explained by its high content of tocopherols and unsaturated fatty acids, low abundance and type of volatile compounds, as well as low level of CV and TPC.

On the whole, the different performance in the frying of camellia oil in the data matrix was revealed: unsaturated fatty acids and tocopherols were consumed to constantly promote the generation of the compounds belonging to TPC, CV, and aroma. Unsaturated fatty acids and tocopherols are the most common nutrients in edible oil, which are directly related to physical and chemical reactions and were the source of quality changes in camellia oil during frying. Under the influence of severe frying conditions, they showed dynamic decomposition. The resulting decomposition products gradually volatilized or accumulated in camellia oil, giving the food a unique fried flavor. On the other hand, their continuous accumulation in oil led to the continuous increase in carbonyl compounds and polar compounds in the oil, which manifested as a gradual increase in CV and TPC. These changes in internal factors and external performance of camellia oil together affect the quality of frying oil.

## 4. Conclusions

In this work, the quality changes in camellia oil during frying were comprehensively evaluated based on multiple perspectives, including oil composition, deterioration indicators, and volatile compounds. It showed that the degradation of unsaturated fatty acids and tocopherols in camellia oil occurred during frying, resulting in a significant increase in CV and TPC. Based on computing the CV and TPC limits, the frying life of camellia oil was predicted to be less than 31.94 h. Furthermore, heat treatment promoted the formation of volatile compounds, from ten before frying to 32 after frying. Aldehydes accumulated continuously during frying, and their abundance was significantly positively correlated with CV. The total abundance of all volatile compounds increased significantly and was positively correlated with TPC during frying. The PCA results confirmed that the quality of camellia oil during frying can be divided into three stages: camellia oil with a heating time of 2.5–7.5 h, 10.0–15.0 h, and 17.5–25.0 h, which corresponded to the degradation acceleration stage, degradation deceleration stage, and degradation stagnation stage of tocopherol, respectively.

Finally, the present study offers valuable insights into the quality changes in camellia oil during frying and can lay a foundation for subsequent research. Due to the practical limitations and complexities, this work cannot comprehensively review the quality changes of multiple types of camellia oil during frying. Further research still needs to focus on verifying the effects of multiple types of frying on the deterioration of camellia oil and taking measures to delay the quality change in camellia oil.

## Figures and Tables

**Table 1:** Change in fatty acid composition during frying.

Time (h)	Fatty Acid (%)						
	Palmitic Acid	Stearic Acid	Oleic Acid	Linoleic Acid	Linolenic Acid	PUFA	TUFA
0.0	7.02 ± 0.08 ^a^	1.53 ± 0.02 ^b^	78.42 ± 0.18 ^a^	11.13 ± 0.05 ^c^	1.91 ± 0.03 ^a^	13.04 ± 0.08 ^b^	91.45 ± 0.10 ^a^
2.5	7.12 ± 0.02 ^a^	1.50 ± 0.01 ^a^	78.74 ± 0.04 ^a^	10.83 ± 0.02 ^d^	1.81 ± 0.00 ^b^	12.64 ± 0.02 ^c^	91.38 ± 0.03 ^a^
5.0	7.13 ± 0.25 ^a^	1.64 ± 0.00 ^cd^	77.83 ± 0.29 ^b^	11.49 ± 0.05 ^a^	1.91 ± 0.02 ^a^	13.40 ± 0.07 ^a^	91.23 ± 0.25 ^a^
7.5	7.93 ± 0.53 ^b^	1.66 ± 0.02 ^d^	77.34 ± 0.43 ^de^	11.22 ± 0.08 ^b^	1.85 ± 0.00 ^b^	13.07 ± 0.08 ^b^	90.41 ± 0.51 ^b^
10.0	8.22 ± 0.19 ^bc^	1.64 ± 0.04 ^c^	77.69 ± 0.34 ^bcd^	10.74 ± 0.11 ^e^	1.71 ± 0.00 ^c^	12.45 ± 0.11 ^d^	90.14 ± 0.23 ^bc^
12.5	8.32 ± 0.15 ^c^	1.74 ± 0.01 ^e^	77.24 ± 0.03 ^e^	10.90 ± 0.06 ^d^	1.80 ± 0.07 ^b^	12.70 ± 0.13 ^c^	89.94 ± 0.15 ^cd^
15.0	8.48 ± 0.19 ^c^	1.79 ± 0.01 ^f^	77.49 ± 0.15 ^bcde^	10.59 ± 0.06 ^f^	1.66 ± 0.01 ^d^	12.24 ± 0.06 ^e^	89.73 ± 0.19 ^d^
17.5	9.03 ± 0.21 ^d^	1.80 ± 0.01 ^f^	77.34 ± 0.26 ^de^	10.26 ± 0.05 ^g^	1.57 ± 0.01 ^e^	11.83 ± 0.04 ^f^	89.17 ± 0.22 ^e^
20.0	9.34 ± 0.22 ^de^	1.85 ± 0.00 ^g^	77.40 ± 0.13 ^cde^	9.93 ± 0.08 ^h^	1.47 ± 0.04 ^f^	11.40 ± 0.11 ^g^	88.81 ± 0.23 ^f^
22.5	9.41 ± 0.15 ^e^	1.90 ± 0.00 ^h^	77.73 ± 0.17 ^bc^	9.56 ± 0.03 ^i^	1.40 ± 0.01 ^g^	10.96 ± 0.02 ^h^	88.69 ± 0.15 ^f^
25.0	9.98 ± 0.38 ^f^	1.93 ± 0.01 ^i^	77.58 ± 0.41 ^bcde^	9.20 ± 0.01 ^j^	1.30 ± 0.03 ^h^	10.51 ± 0.04 ^i^	88.09 ± 0.37 ^g^

PUFA, polyunsaturated fatty acids; TUFA, total unsaturated fatty acids. Different superscript letters in the same column indicate significant differences (*p* < 0.05).

**Table 2:** The fitting results of fatty acids’ change with time during frying.

Fatty Acid	Slope	R^2^
Palmitic acid	1.58	0.965
Stearic acid	1.07	0.957
Oleic acid	−0.06	0.284
Linoleic acid	−0.51	0.724
Linolenic acid	−1.07	0.863
PUFA	−0.59	0.762
TUFA	−0.14	0.974

**Table 3:** Change in content of tocopherols during frying.

Time (h)	Tocopherol (mg/100 g)					Loss Rate (%)
	α	β	γ	δ	Total	
0.0	8.19 ± 0.37 ^a^	0.06 ± 0.00 ^a^	4.39 ± 0.20 ^a^	0.13 ± 0.01 ^ab^	12.77 ± 0.58 ^a^	0.00
2.5	7.57 ± 0.41 ^b^	0.04 ± 0.00 ^b^	3.98 ± 0.22 ^b^	0.12 ± 0.01 ^ab^	11.71 ± 0.62 ^b^	8.34
5.0	5.87 ± 0.18 ^c^	0.03 ± 0.00 ^c^	3.11 ± 0.10 ^c^	0.12 ± 0.00 ^ab^	9.13 ± 0.28 ^c^	28.41
7.5	3.48 ± 0.02 ^d^	0.02 ± 0.00 ^d^	2.10 ± 0.00 ^d^	0.12 ± 0.00 ^a^	5.72 ± 0.11 ^d^	55.19
10.0	1.65 ± 0.01 ^e^	0.01 ± 0.00 ^e^	1.17 ± 0.04 ^e^	0.11 ± 0.01 ^bc^	2.94 ± 0.06 ^e^	77.05
12.5	0.19 ± 0.01 ^f^	0.01 ± 0.00 ^f^	0.57 ± 0.02 ^f^	0.10 ± 0.01 ^bc^	0.87 ± 0.03 ^f^	93.23
15.0	0.08 ± 0.00 ^f^	-	0.23 ± 0.02 ^g^	0.09 ± 0.00 ^bcd^	0.40 ± 0.01 ^g^	96.89
17.5	0.08 ± 0.00 ^f^	-	0.06 ± 0.00 ^h^	0.08 ± 0.00 ^bcd^	0.22 ± 0.00 ^g^	98.35
20.0	0.07 ± 0.00 ^f^	-	0.05 ± 0.00 ^h^	0.06 ± 0.00 ^cd^	0.18 ± 0.00 ^g^	98.51
22.5	0.07 ± 0.00 ^f^	-	0.05 ± 0.00 ^h^	0.06 ± 0.00 ^cd^	0.18 ± 0.00 ^g^	98.60
25.0	0.06 ± 0.00 ^f^	-	0.04 ± 0.00 ^h^	0.05 ± 0.00 ^d^	0.15 ± 0.00 ^g^	98.84

“-”, not detected. Different superscript letters in the same column indicate significant differences (*p* < 0.05).

**Table 4:** The volatile compounds identified in the headspace of the oil samples.

No.	RT (min)	RI	RIr	Volatile Compounds	Peak Area of Volatile Compounds Identified in Oil Samples with Different Heating Times										
					0.0	2.5	5.0	7.5	10.0	12.5	15.0	17.5	20.0	22.5	25.0
Alcohol															
1	1.72	<600	489	Ethanol	56180 ± 114 ^f^	7788 ± 648 ^a^	8181 ± 151 ^a^	8374 ± 270 ^a^	8523 ± 565 ^ab^	10320 ± 307 ^b^	12870 ± 647 ^c^	12890 ± 223 ^c^	15041 ± 753 ^d^	16125 ± 795 ^e^	16402 ± 1027 ^e^
2	1.78	<600	621	2-Methylpropan-1-ol	12804 ± 43 ^a^	152038 ± 5117 ^f^	224495 ± 2207 ^h^	131626 ± 3668 ^c^	146445 ± 7544 ^ef^	121075 ± 3456 ^b^	192245 ± 962 ^g^	143751 ± 2456 ^de^	194646 ± 1219 ^g^	137312 ± 5779 ^cd^	147082 ± 4947 ^ef^
3	3.10	714	686	Pent-1-en-3-ol	-	6959 ± 66 ^a^	9081 ± 45 ^c^	6804 ± 197 ^a^	7327 ± 91 ^a^	8243 ± 708 ^b^	10943 ± 212 ^de^	10706 ± 212 ^d^	12497 ± 627 ^f^	12146 ± 295 ^f^	11285 ± 143 ^e^
4	5.03	795	764	Pentan-1-ol	-	2771 ± 101 ^a^	3985 ± 165 ^b^	4543 ± 189 ^b^	4655 ± 250 ^b^	5840 ± 420 ^c^	7408 ± 456 ^d^	8355 ± 293 ^e^	9730 ± 65 ^f^	10033 ± 81 ^f^	10371 ± 687 ^f^
5	13.60	997	965	Heptan-1-ol	-	328 ± 13 ^a^	546 ± 28 ^b^	753 ± 37 ^bc^	728 ± 29 ^c^	971 ± 4 ^d^	1217 ± 47 ^de^	1356 ± 53 ^de^	1661 ± 119 ^f^	1515 ± 32 ^f^	1423 ± 47 ^ef^
6	17.90	1097	1070	Octan-1-ol	-	228 ± 1 ^a^	433 ± 43 ^b^	482 ± 52 ^ab^	772 ± 25 ^c^	908 ± 39 ^d^	897 ± 23 ^d^	920 ± 76 ^d^	1239 ± 94 ^e^	1139 ± 79 ^e^	1330 ± 40 ^e^
Aldehyde															
7	2.03	619	601	Butanal	907 ± 27 ^abc^	1380 ± 137 ^bc^	885 ± 68 ^abc^	868 ± 95 ^abc^	657 ± 10 ^a^	881 ± 69 ^abc^	1208 ± 7 ^bc^	852 ± 44 ^ab^	1309 ± 180 ^bc^	1115 ± 166 ^abc^	1521 ± 57 ^c^
8	2.70	692	657	(*E*)-But-2-enal	-	2446 ± 10 ^a^	3728 ± 161 ^b^	2710 ± 185 ^a^	3405 ± 134 ^b^	4275 ± 106 ^c^	6762 ± 450 ^d^	7383 ± 45 ^e^	9070 ± 272 ^g^	8695 ± 35 ^f^	8697 ± 298 ^f^
9	2.88	704	664	2-Methylbutanal	5145 ± 112 ^a^	8754 ± 142 ^e^	8810 ± 374 ^e^	8056 ± 437 ^d^	5652 ± 301 ^a^	6167 ± 126 ^c^	7106 ± 234 ^c^	5365 ± 231 ^a^	6435 ± 514 ^bc^	5826 ± 389 ^ab^	8315 ± 344 ^de^
10	3.34	724	701	Pentanal	6017 ± 55 ^a^	51617 ± 1306 ^b^	80296 ± 424 ^g^	62084 ± 905 ^c^	70645 ± 380 ^e^	66498 ± 349 ^d^	93133 ± 677 ^i^	77233 ± 523 ^f^	101482 ± 392 ^j^	84322 ± 329 ^h^	94284 ± 2926 ^i^
11	4.65	779	759	(*E*)-Pent-2-enal	-	1172 ± 121 ^a^	1739 ± 159 ^b^	1775 ± 46 ^b^	2062 ± 164 ^b^	2270 ± 20 ^c^	3673 ± 126 ^d^	4807 ± 162 ^e^	6357 ± 397 ^f^	7313 ± 12 ^g^	7387 ± 142 ^g^
12	6.14	824	802	Hexanal	1477 ± 96 ^a^	31899 ± 205 ^b^	45520 ± 365 ^d^	39555 ± 1714 ^c^	48037 ± 550 ^d^	56662 ± 152 ^e^	84428 ± 2094 ^f^	99801 ± 2237 ^g^	128433 ± 2339 ^h^	153853 ± 1838 ^i^	163206 ± 5931 ^j^
13	8.30	877	864	(*E*)-Hex-2-enal	-	885 ± 50 ^a^	1340 ± 70 ^b^	1412 ± 93 ^b^	1471 ± 146 ^bc^	1647 ± 109 ^cd^	1891 ± 96 ^de^	2078 ± 149 ^ef^	2239 ± 282 ^f^	2366 ± 56 ^f^	2375 ± 57 ^f^
14	10.45	927	903	Heptanal	-	1943 ± 195 ^a^	2819 ± 79 ^ab^	3221 ± 263 ^c^	2992 ± 121 ^bc^	3581 ± 196 ^c^	4424 ± 390 ^d^	5018 ± 225 ^e^	6314 ± 126 ^f^	6419 ± 288 ^f^	7223 ± 427 ^g^
15	12.90	981	956	(*E*)-Hept-2-enal	-	4173 ± 127 ^a^	5878 ± 313 ^b^	6040 ± 530 ^b^	7206 ± 240 ^c^	8782 ± 263 ^d^	10015 ± 18 ^e^	10215 ± 192 ^e^	11303 ± 392 ^f^	11387 ± 329 ^f^	10985 ± 771 ^f^
16	15.00	1029	1003	Octanal	-	2150 ± 191 ^a^	2276 ± 188 ^ab^	2426 ± 404 ^ab^	2954 ± 157 ^bc^	3517 ± 270 ^c^	4702 ± 231 ^d^	4877 ± 291 ^de^	5448 ± 142 ^ef^	5603 ± 235 ^fg^	5432 ± 65 ^g^
17	15.30	1036	1015	(2*E*,4*E*)-Hepta-2,4-dienal	-	5223 ± 464 ^a^	6817 ± 234 ^cd^	6820 ± 234 ^cd^	6896 ± 389 ^cd^	6202 ± 77 ^b^	7383 ± 163 ^d^	6866 ± 77 ^cd^	6538 ± 543 ^bc^	5595 ± 235 ^a^	5519 ± 223 ^a^
18	17.30	1083	1064	(*E*)-Oct-2-enal	-	702 ± 47 ^a^	810 ± 58 ^ab^	847 ± 185 ^bc^	1283 ± 61 ^d^	1219 ± 107 ^cd^	1286 ± 135 ^cd^	1353 ± 63 ^d^	1593 ± 118 ^e^	1671 ± 39 ^ef^	1773 ± 108 ^f^
19	19.30	1132	1104	Nonanal	-	5285 ± 230 ^a^	6712 ± 459 ^b^	7251 ± 636 ^b^	8761 ± 236 ^cd^	8318 ± 676 ^c^	8893 ± 130 ^cd^	9012 ± 693 ^cd^	8734 ± 244 ^cd^	10273 ± 652 ^d^	9294 ± 34 ^d^
20	21.50	1187	1165	(*E*)-Non-2-enal	-	-	441 ± 18 ^a^	528 ± 20 ^ab^	664 ± 77 ^cd^	599 ± 46 ^bc^	724 ± 22 ^cd^	916 ± 10 ^de^	854 ± 9 ^ef^	1125 ± 64 ^f^	1026 ± 57 ^ef^
21	25.50	1294	1263	(*E*)-Dec-2-enal	-	665 ± 48 ^a^	1304 ± 67 ^b^	1841 ± 19 ^c^	2272 ± 95 ^c^	2833 ± 243 ^d^	3191 ± 153 ^e^	3908 ± 82 ^g^	3949 ± 78 ^g^	3699 ± 69 ^f^	3537 ± 156 ^f^
22	26.70	1327	1316	(2*E*,4*E*)-Deca-2,4-dienal	-	292 ± 27 ^a^	479 ± 104 ^b^	561 ± 48 ^b^	679 ± 40 ^bc^	791 ± 51 ^cd^	824 ± 40 ^cd^	797 ± 143 ^cd^	979 ± 83 ^d^	869 ± 64 ^cd^	799 ± 70 ^cd^
23	27.50	1350	1360	(*E*)-Undec-2-enal	-	646 ± 0 ^a^	2019 ± 130 ^b^	2785 ± 172 ^c^	3161 ± 159 ^cd^	3773 ± 254 ^e^	3702 ± 243 ^e^	4449 ± 268 ^f^	3871 ± 227 ^e^	3424 ± 363 ^e^	3146 ± 180 ^de^
24	29.10	1396	1420	(2*E*,4*E*)-Undeca-2,4-dienal	-	-	402 ± 18 ^a^	678 ± 17 ^b^	1152 ± 96 ^c^	1762 ± 106 ^d^	1928 ± 177 ^e^	2332 ± 90 ^e^	2185 ± 129 ^e^	2117 ± 1 ^e^	1969 ± 62 ^e^
Other volatile compounds															
25	1.89	604	559	Methyl acetate	3737 ± 121 ^d^	6276 ± 330 ^h^	5754 ± 166 ^g^	6162 ± 269 ^h^	2735 ± 122 ^b^	4323 ± 286 ^e^	4749 ± 242 ^f^	2082 ± 98 ^a^	1952 ± 71 ^a^	1793 ± 4 ^a^	3327 ± 140 ^c^
26	2.34	653	612	Ethyl acetate	10241 ± 89 ^c^	17939 ± 456 ^i^	16614 ± 90 ^h^	15317 ± 49 ^g^	9964 ± 413 ^c^	11501 ± 188 ^d^	12369 ± 403 ^e^	9206 ± 753 ^b^	9775 ± 177 ^bc^	8461 ± 332 ^a^	13005 ± 428 ^f^
27	2.11	628	600	Acetic acid	-	-	1354 ± 451 ^ab^	568 ± 80 ^a^	1973 ± 134 ^b^	1198 ± 67 ^ab^	2085 ± 220 ^b^	4878 ± 642 ^c^	8106 ± 144 ^d^	11466 ± 404 ^e^	11731 ± 117 ^e^
28	2.23	641		Hexane	2247 ± 96 ^a^	7311 ± 119 ^b^	9417 ± 296 ^cd^	8766 ± 293 ^c^	7998 ± 113 ^b^	8321 ± 387 ^b^	9968 ± 709 ^d^	11057 ± 383 ^e^	14223 ± 557 ^f^	14014 ± 167 ^f^	16806 ± 597 ^g^
29	2.98	709		Heptane	6742 ± 29 ^ab^	11206 ± 610 ^f^	11317 ± 830 ^f^	10119 ± 237 ^e^	6750 ± 321 ^ab^	8508 ± 674 ^c^	8820 ± 420 ^cd^	6001 ± 300 ^a^	6948 ± 307 ^b^	6358 ± 335 ^ab^	9446 ± 552 ^de^
30	11.80	957	941	Butylcyclopentane	-	1300 ± 89 ^a^	1735 ± 124 ^c^	1473 ± 101 ^b^	1852 ± 106 ^cd^	2106 ± 143 ^cd^	2272 ± 121 ^e^	1888 ± 93 ^cd^	2283 ± 169 ^e^	2035 ± 44 ^de^	2023 ± 143 ^d^
31	14.40	1015	993	2-Pentylfuran	-	2404 ± 205 ^a^	2824 ± 60 ^bc^	2601 ± 149 ^ab^	2640 ± 273 ^ab^	2694 ± 56 ^ab^	3058 ± 216 ^c^	3128 ± 65 ^bc^	3438 ± 339 ^d^	3992 ± 116 ^e^	4328 ± 89 ^e^
32	14.65	1021	1001	2-Ethyl-6-methylpyrazine	-	1359 ± 115 ^a^	1698 ± 105 ^b^	1787 ± 46 ^b^	1638 ± 60 ^b^	1741 ± 112 ^b^	1799 ± 176 ^b^	1743 ± 116 ^b^	1837 ± 180 ^b^	1542 ± 100 ^b^	1534 ± 84 ^b^

The volatile compounds are arranged in order of their retention time (RT) within each classification. The retention index (RI) was calculated for the SH-RxiTM-5SilMS capillary column. The volatile compounds were identified by comparing the mass spectra with those in the NIST 17 Mass Spectral Library and comparing the RI with the RI in references (RIr) with similar phase columns used for the study of frying oil. “-”, not detected. Different superscript letters in the same column indicate significant differences (*p* < 0.05).

## Data Availability

Data are contained within the article.
